# Influence of Operational Strategies for the Recovery of Magnesium Hydroxide from Brines at a Pilot Scale

**DOI:** 10.1021/acs.iecr.2c02935

**Published:** 2022-10-04

**Authors:** Carmelo Morgante, Fabrizio Vassallo, Giuseppe Battaglia, Andrea Cipollina, Fabrizio Vicari, Alessandro Tamburini, Giorgio Micale

**Affiliations:** † Dipartimento di Ingegneria, Università degli Studi di Palermo (UNIPA), viale delle Scienze Ed. 6, Palermo90128, Italy; ‡ ResourSEAs SrL, viale delle Scienze Ed. 16, Palermo90128, Italy

## Abstract

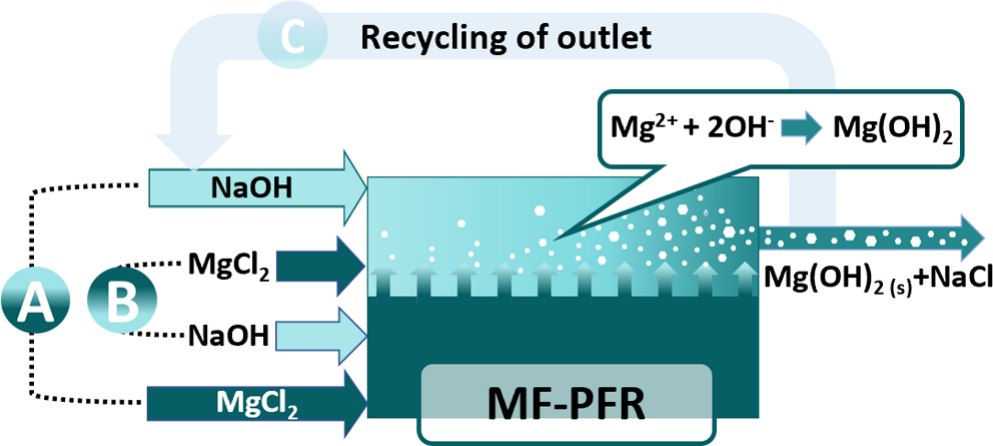

The continuous depletion of minerals caused by land mining and the increase in their demand have pushed the development of novel sustainable technological processes for mineral recovery from unconventional sources. In this context, magnesium (Mg) has gained considerable attention for its peculiar properties and high relevance of its compounds, such as magnesium hydroxide, Mg(OH)_2_. In the present work, the influence of several operating conditions on the Mg(OH)_2_ precipitation process was thoroughly investigated by adopting a novel multiple feed-plug flow reactor. The influence of (i) initial Mg^2+^ concentrations in the feed stream; (ii) brine and alkaline flow rates; and (iii) the product recycling strategy (seeded crystallization) was considered. The results marked the possibility of improving sedimentation and filterability properties of Mg(OH)_2_ suspensions by adopting the recycling strategy to overcome industrial issues associated with the production of Mg(OH)_2_ suspensions using NaOH solutions.

## 1 Introduction

Over the past couple of decades, gradual depletion of minerals by land mining and the increase of their demand have driven the European Union to define 30 “critical” raw materials (CRMs).^1^ The recovery of such materials has been one of the main focuses of EU’s Green Deal:^2^ an action plan aimed at the development of sustainable technological processes, reducing both environmental pollution and economic dependence from other nations.

Among the CRMs, magnesium (Mg) has lately gained particular attention within the European economy. As a matter of fact, one of its main compounds, magnesium hydroxide [Mg(OH)_2_], is nowadays widely employed in several industrial sectors due to its intriguing and unique chemical and physical properties.^3^ Among the applications, its use in the pharmaceutical field, refractories, wastewater treatment industry, and desulfurization of gases is worth mentioning.^4−6^ Moreover, Mg(OH)_2_ is employed by calcination for the production of magnesium oxide (MgO),^7^ and it has captured greater interest due to its fundamental use for safety and protection purposes.^8^ More specifically, under fire conditions, it degrades at high temperatures (around 350 °C) producing water vapor that forms an envelope around the flame excluding air and diluting flammable gases. Such behavior makes Mg(OH)_2_ an optimal toxic-free flame retardant for use in polymeric materials.^9,10^

In satisfying the current global demand of Mg(OH)_2_, recent years have seen a shift of exploiting the mineral source from mines to seawater. Such a trend has been primarily induced by the steady depleting availability of high-grade mineral deposits that are easily accessible, leaving more of the low-grade ore found deeper in the lands. As the ore grade diminishes, production costs such as water and energy costs tend to increase.^11^ Furthermore, features like water shortages and energy requirements, accompanied by lasting environmental damage, make the mining industry even more unattractive.^12,13^ For this reason, seawater has become more and more appealing as an alternative mineral source. However, it is not a ground-breaking discovery that seawater contains many elements present in the periodic table, of which Mg is the second most abundant mineral after sodium (Na),^8^ comprising approximately 15% of the total salt.^14^ Therefore, seawater can be considered as an extremely abundant source for Mg recovery.^8,15,16^ However, seawater exploitation is not unconventional and has been applied for Mg recovery for decades.^17−19^ Nowadays, there are a number of facilities around the world that produce Mg-based compounds from seawater, for example, in Ireland, Japan, Norway, and the USA.^14^ Nevertheless, relatively low concentrations and high expenses associated with the extraction methods have mitigated mineral production from seawater.

A possible solution to overcome such issues and promote more sustainable extraction techniques could be the use of desalination brine as higher Mg^2+^ concentrations are present.^20−25^ The so-called “brine valorization” not only ensures the production of high-value minerals but also can reduce both the cost of water produced by desalination plants and the environmental impact of brine discharge.^26−29^ This idea, based on a more sustainable management of brine, brings life to the concept of circular economy.

Furthermore, the removal of Mg(OH)_2_ from brines is a win-win situation not only for its aforementioned prospective applications but also for the fact of withdrawing contemporarily a scaling compound that could compromise membrane-based, evaporative, desalination technologies and brine concentrators within zero liquid discharge (ZLD) systems.^30−32^

Despite the source from which Mg is recovered, various methods for Mg(OH)_2_ production have been introduced in the past in the scientific literature such as hydration of MgO, precipitation of salt with an alkaline solution, electrolysis of an aqueous Mg salt solution,^33^ and sol–gel technique.^34^ However, the majority of works present in the literature concern reactive chemical precipitation. The main reasons for this are simplicity of the method, low-cost apparatuses, and ease of commercialization.^35^ Various alkaline reagents have been employed to induce Mg(OH)_2_ precipitation. Ammonia has been employed as the alkaline reactant.^20,36^ More specifically, Mohammad et al.^20^ recovered Mg from desalination reject brine by adopting ammonium hydroxide (NH_4_OH) and achieving >95% pure Mg(OH)_2_ products. Experiments also highlight the difficulty in achieving total Mg conversion in the process, with 97% being the highest obtained recovery.

A very safe and also low-cost reactant is, on the other hand, calcium hydroxide [Ca(OH)_2_] or slaked lime. The low cost of such reactant led Dow Chemical Company to patent in 1943 a process to recover Mg from seawater by precipitation with lime. Several works^21,24,25,37^ have attempted to employ Ca(OH)_2_, achieving high recoveries of Mg but low purities of the final product (typically below 80% and up to 91% when screening operations to lime were applied). Low purities are due to the presence of impurities in lime. The presence of carbonates or calcium ions in the Mg source can lead to the co-precipitation of calcium sulfate, Ca(OH)_2_, and calcium carbonate during the production of Mg(OH)_2_. Industrially, high purity Mg(OH)_2_ required to produce pure MgO up to 97%^38^ via calcination can be achieved by using low-impurity raw stones and adequate calcination conditions for the production of highly pure lime solutions. Furthermore, several pre-treatments are required for the decarbonation or removal of suspended particles in the Mg source. An innovative alternative has been recently proposed by La Corte et al.^39^ and Vassallo et al.^40^ who recently presented a novel membrane crystallizer called “CrIEM” in which brine enters in contact with a low-cost reactant [Ca(OH)_2_] by means of an anionic exchange membrane, promoting the precipitation of high-purity Mg(OH)_2_ particles. This technology, however, is still at the lab scale and could be still far away from a possible future industrial application due to the use of expensive membranes, thus leading to high capital costs.

The danger brought about by the use of ammonia and low purity Ca(OH)_2_ has driven many researchers to investigate Mg(OH)_2_ precipitation performances by means of sodium hydroxide (NaOH). Casas et al.^22^ demonstrated how the use of NaOH allowed achieving higher purity Mg(OH)_2_ than those achieved by means of sodium carbonate (Na_2_CO_3_). Song et al.^17,18^ also achieved high-purity Mg(OH)_2_ particles from concentrated saline solutions. By means of a mixed suspension mixed product removal crystallizer, spherical particles with purities higher than 99% were achieved, characterized by an average particle size distribution (PSD) ranging from 6 to 30 μm. However, a drawback consisted in the high tendency of particle agglomeration forming gelatinous suspensions, leading to difficulty in filtration.^41^ Turek and Gnot^42^ recovered Mg(OH)_2_ as a byproduct by means of NaOH from hard coal mine brine. The authors reported that if an excess of hydroxide ions was maintained during crystallization, the sedimentation speed would be slower, and filtration would be more difficult than the case characterized by an excess of Mg^2+^ ions. Lee and Lim^43^ proposed a multi-step reactive process for recycling magnesium chloride (MgCl_2_) from industrial brines. Once sulfuric acid was added to the brine to precipitate calcium ions, NaOH was then added producing Mg(OH)_2_ with a purity of 98% and a hexagonal flat platelet structure. Additives such as carboxymethyl cellulose and sodium stearate were added, which halve the sedimentation times and achieve a crystal size of 5 μm and a purity of 99.5%. Henrist et al.^33^ investigated how the use of NaOH or NH_4_OH would affect the size, shape, and level of agglomeration of Mg(OH)_2_ crystals produced from artificial brines. The use of NaOH led to cauliflower-shaped globular agglomerates at 60 °C, while employing ammonia resulted in more resistant platelet-shaped particles, as also reported by Li et al.^44^ Moreover, the influence of the operating temperature on the characteristics of the final product was examined. Higher temperatures led to smaller crystals that agglomerate more. Recent work performed by Jarosinski et al.^45^ concerned the introduction of a new method in which the reaction with NaOH was followed by washing with a 25% ammonia solution and acetone. Such a procedure enabled achieving a product with a high specific surface area of 100 m^2^/g (higher than the one required for flame retardant purposes <10 m^2^/g). Cipollina et al.^46^ carried out an experimental campaign with semi-batch and continuous reactors. Higher concentrations of the alkaline reactant and Mg^2+^ allow the formation of larger particles. Purity was between 98 and 100% in most experimental runs.

Based on all works mentioned previously, advantages and disadvantages of possible reactants for Mg(OH)_2_ precipitation can be summarized as follows: (i) NH_4_OH leads to not only highly pure hexagonal Mg(OH)_2_ particles but also low Mg^2+^ conversion and the production of byproducts (e.g., ammonia), which is considered dangerous when the slurry is further employed in electrolytic processes;^42^ (ii) the use of lime causes the production of a low-purity Mg(OH)_2_ product, despite its low cost; (iii) NaOH allows the production of highly pure Mg(OH)_2_ products with 100% conversion of Mg^2+^.^46^ On the other hand, NaOH is expensive and leads to the precipitation of gelatinous suspensions that are difficult to be sedimented and filtered.^46^

Therefore, within the framework of attempting to overcome the previous issues associated with NaOH employment (slow sedimentation, slow filtration, and production of small particles), the present work aims at investigating the influence of different operating conditions and process strategies focusing on the Mg(OH)_2_ precipitation process conducted using the novel multiple feed-plug flow reactor (MF-PFR) designed by ResourSEAs SrL and recently introduced by Vassallo et al.^47,48^ at the Brine Excellence Centre of the University of Palermo. To the best of the authors’ knowledge, it is the first unstirred reactive crystallizer that has been developed at a pilot scale and believed to produce Mg(OH)_2_ from waste industrial brines. The MF-PFR is a modular reactor that can be easily scaled up with respect to classical batch stirred reactors whose design at large scales poses severe issues as the volume of the reactor increases.^49^ The MF-PFR was initially tested by Vassallo et al.^47^ to selectively recover Mg(OH)_2_ and Ca(OH)_2_ at controlled pH values from spent brines within the water softening industry. Specifically, the MF-PFR was employed to treat the retentate of the nanofiltration unit processing the spent brine from the industrial water production plant of Evides Industriewater B.V. (Rotterdam). The authors conducted an extensive experimental campaign aimed at demonstrating the stability and robustness of the prototype at different inlet flow rates and initial brine composition. The performances of the MF-PFR were assessed on the basis of Mg(OH)_2_ and Ca(OH)_2_ purity without assessing the influence of such operating parameters on the properties of the produced slurries, for example, sedimentation rate, filtration rate, and PSDs. Results marked the possibility of achieving high values of mineral recovery: 100 and 97% for magnesium and calcium hydroxides, respectively. High purity of these final products (>98%) was another successful accomplishment.^50^

In the present work, the focus is on the assessment of the influence of different operating conditions on the sedimentation rate, filtration rate, and granulometry characteristics of final Mg(OH)_2_ products obtained by adopting the same MF-PFR as functions of (i) the initial Mg^2+^ brine concentration, (ii) the brine/NaOH flow rate at a fixed initial Mg^2+^ concentration, and (iii) the possibility of recycling part of the product to induce a seeded precipitation process. Two Mg^2+^-containing brine scenarios were considered: (i) a brine mimicking the Mg^2+^ concentration of a real brine exiting a nanofiltration unit treating seawater^51^ and (ii) a brine mimicking the Mg^2+^ concentration of a real brine exiting a typical Mediterranean saltwork.^46^ Furthermore, different reactor configurations were investigated. The present work will be of great help to support the evolution of mineral recovery in circular economy schemes, providing essential information for the design of Mg(OH)_2_ industrial reactors. In particular, the novel aspects investigated here will aid in overcoming the typical sedimentation and filterability issues associated with the precipitation of Mg(OH)_2_ via NaOH solutions.

### 1.1 Concept of Mg(OH)_2_ Recovery via a Novel Reactive Crystallizer

**Figure 1:**
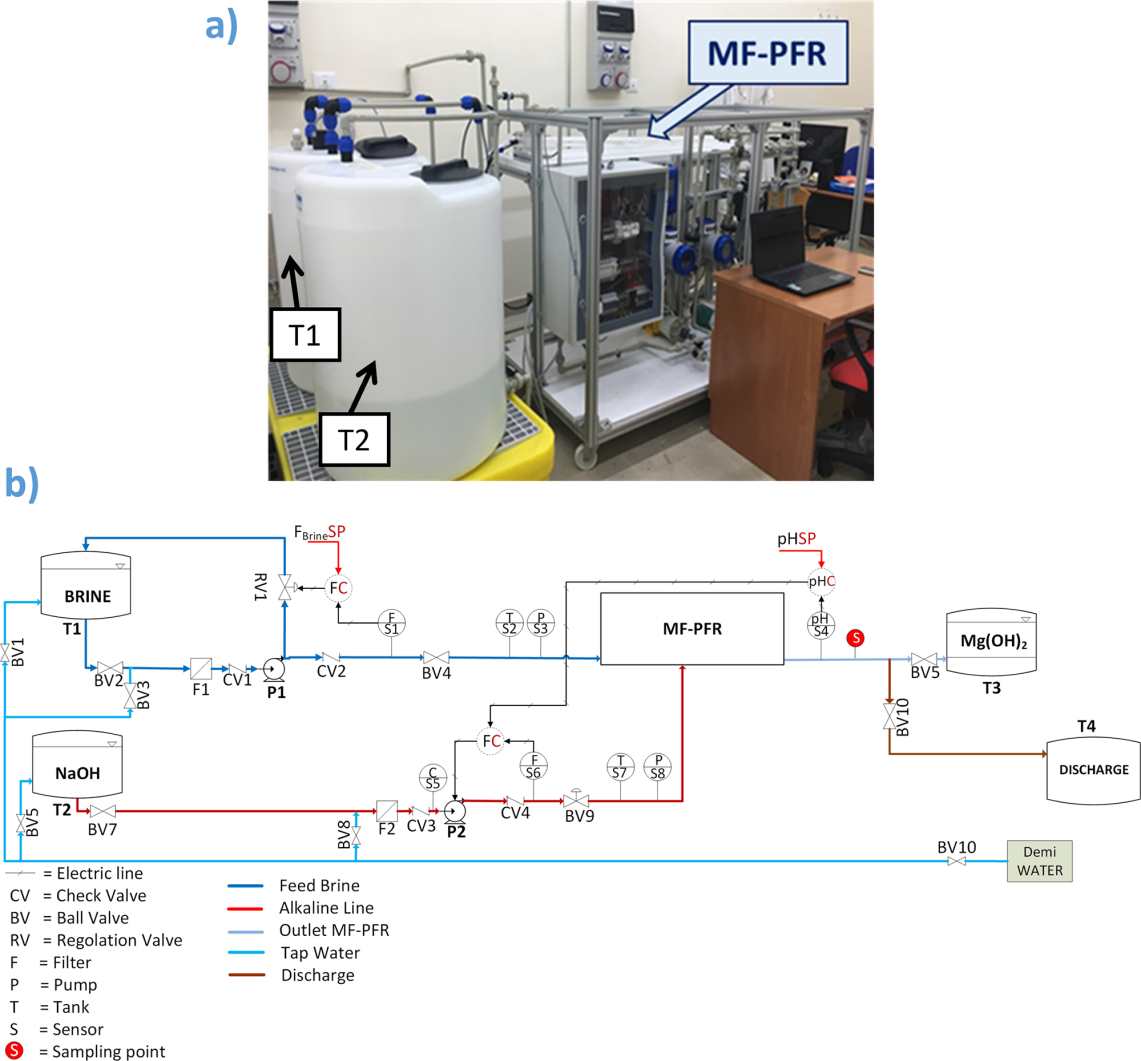
(a) MF-PFR prototype developed at the Brine Excellence Centre satellite laboratory of the University of Palermo^47^ and (b) simplified P&ID of the MF-PFR prototype. Figure 1a was adapted from Vassallo et al.^47^

The novel reactive crystallizer under investigation in this work is the MF-PFR introduced by Vassallo et al.^47,48^Figure 1 presents (a) a picture of the developed MF-PFR pilot plant and (b) a simplified P&ID describing all the features of the plant.

The experimental setup (see Figure 1a,b) is made of the MF-PFR pilot unit consisting in an aluminum structural skid incorporating the reactor itself with all its auxiliary units. Pumps P1 and P2 are employed to feed the brine and NaOH solution, respectively, into the MF-PFR from the two 200 L cylindrical tanks in high-density polyethylene (HDPE), labeled T1 and T2. Along the two feed lines, pressure, temperature, and flow rate are measured for both solutions through pressure transducers P/S3-8 (OPTIBAR 1010C, KROHNE, and VEGABAR14, VEGA), temperature sensors T/S2-7 (TRA-C20, KROHNE), and magnetic induction flow-meters F/S1-6 (OPTIFLUX 4300 C, KROHNE). The conductivity of the alkaline solution is also monitored via the sensor C/S5 (IND1000, MAC100, KROHNE). The brine flow rate is adjusted by controlling the percentage of the opening section of the RV1 valve. A pH-meter (PH 8320, KROHNE) is employed to monitor the outlet slurry pH, which is adjusted by varying the alkaline flow rate through a cascade control. The produced Mg(OH)_2_ slurry is then stored in a 500 L cylindrical tank in HDPE, labeled T3. A further tank (T4) is also employed to store (i) the cleaning solutions discharged and (ii) the slurry produced during the startup of the pilot plant. All in all, to monitor and control the desired parameters of the prototype, a control panel was developed in LabVIEW software.

**Figure 2:**
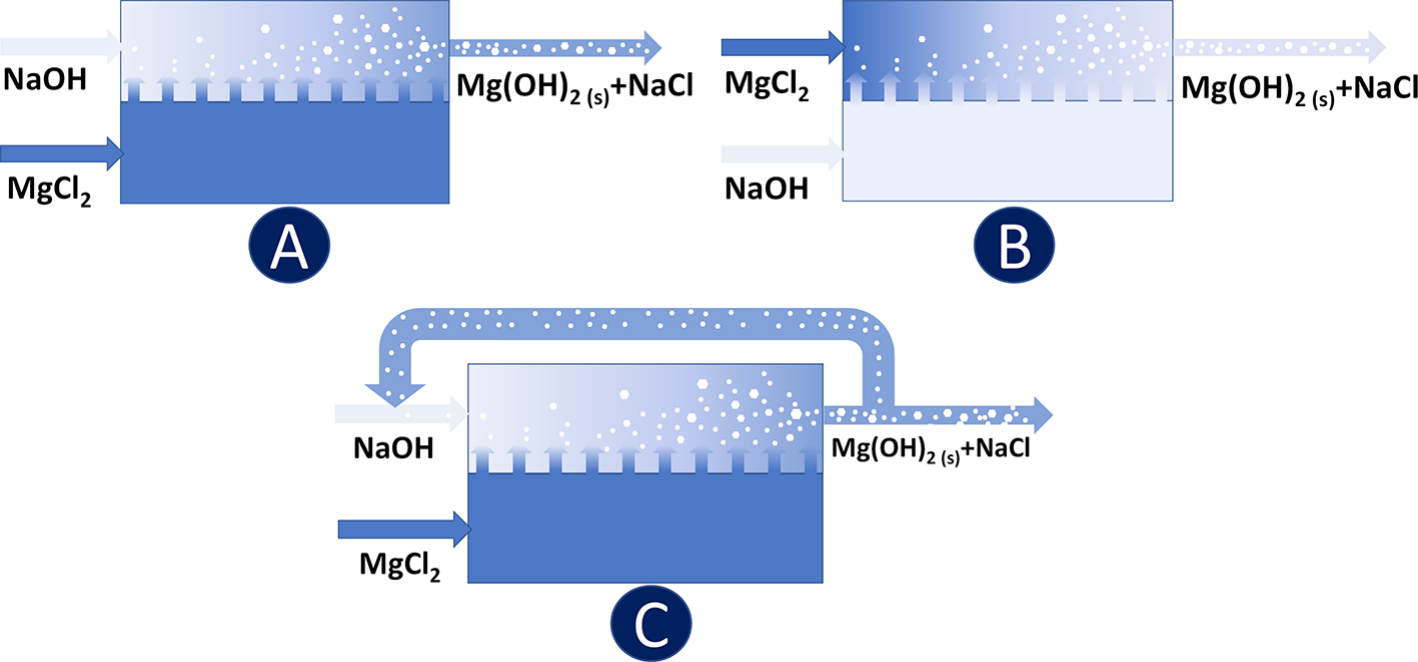
Conceptual schemes of Mg(OH)_2_ precipitation within the MF-PFR made of two adjacent compartments: (a) brine solution is injected into the NaOH solution; (b) NaOH solution is injected into the brine; (c) the same strategy as (a) but the Mg(OH)_2_ slurry is recycled and partially mixed with fresh alkaline solution.

As far as the MF-PFR reactor is concerned, it consists of two adjacent volumes hydraulically connected. The feed (a synthetic solution mimicking the Mg^2+^ concentration in waste brine) is injected into one of the compartments, whereas an aqueous solution of NaOH is fed to the remaining one. As schematically illustrated in Figure 2, the two feed streams enter in contact with each other with a multiple inlet arrangement which is employed in order to favor a better supersaturation homogenization all over the reactor volume. In addition, each inlet is equipped with nozzles, purposely designed to promote the fast mixing of the two streams.^47,48^ When the two reactants meet, Mg^2+^ present in the feed solution (brine) react with the hydroxyl ions (OH^–^) of the alkaline solution, promoting the precipitation of Mg(OH)_2_ according to the following chemical reaction eq 1
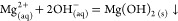
1

The reaction produces a dense white suspension or slurry (due to the color of the precipitated particles). In this work, the performance of the MF-PFR was assessed adopting three different operating strategies that are schematically illustrated in Figure 2. The aim was to understand whether distributing the brine into the alkaline solution or vice versa (the alkaline into the brine) could influence the final product or not (e.g., making a nanoparticle-sized product or a microparticle-sized one, generating a more or less easily filterable product, and so on). Figure 2a shows the standard MF-PFR configuration, here denominated configuration A, in which brine is injected into the alkaline solution, and configuration B (pictured in Figure 2b) where the NaOH is fed in the brine solution. Finally, a third operating configuration (configuration C) was examined, illustrated in Figure 2c. In this case, the Mg(OH)_2_ slurry exiting the reactor was partially mixed with fresh alkaline solution and recycled back to the reactor in order to induce a seeded precipitation and promote a total conversion of Mg ions in the reactor. No reaction takes place downstream the reactor.

The MF-PFR prototype has been accurately designed to best control the reaction pH at which Mg(OH)_2_ precipitation occurs within it. Specifically, all the Mg^2+^ content in a Mg^2+^-containing brine can be assumed to be quantitatively precipitated at the theoretical reaction equilibrium pH value of around 10.4.^40^ For values below 10.4, Mg^2+^ remain in the outlet stream due to incomplete conversion. On the other hand, when values of pH around 12.5–13 are reached, co-precipitation of further ions, according to the composition of the feed stream, can occur, for example, in the presence of calcium ions, Ca(OH)_2_ particles precipitate. Such co-precipitation compromises the purity of Mg(OH)_2_ produced; therefore, the choice of ratio between the flow rate of feed and alkaline reactant is crucial to avoid low purities. The actual possibility of performing pH-controlled Mg(OH)_2_ precipitation tests using the MF-PPR was documented in ref (45).

## 2 Materials and Methods

### 2.1 Overview of the Experimental Campaign

In order to identify how operating conditions of the MF-PFR influences the production of Mg(OH)_2_ in terms of PSD, filterability, and sedimentation rate, an extensive experimental campaign was carried out. To this aim, six tests were performed, which are listed in Table 1. In all tests, synthetic brines containing only MgCl_2_ were employed. Furthermore, the concentration of NaOH was fixed to 1 M in all cases for comparative purposes. The flow rate of the NaOH solution for each test, however, was varied within a certain range to reach final Mg(OH)_2_ slurry pH values of 10.1, 10.4, and 12.

**Table 1:** Main Nominal Operating Conditions of Experimental Tests

	brine concentration						
Test	Mg^2+^[mol/L]	Cl^–^[mol/L]	NaOH conc. [mol/L]	brine flow rate [L/min]	NaOH flow rate range [L/min]	conf^a^	comparison test and purpose
1	0.240 ± 0.005	0.480 ± 0.005	1.00 ± 0.03	0.66 ± 0.04	0.12 ± 0.08–0.38 ± 0.05	A	
2	1.00 ± 0.02	2.00 ± 0.02	1.00 ± 0.03	0.66 ± 0.04	0.59 ± 0.04–1.10 ± 0.03	A	test 1. Initial Mg^2+^ conc.
3	0.240 ± 0.005	0.480 ± 0.005	1.00 ± 0.03	2.00 ± 0.08	0.38 ± 0.06–0.80 ± 0.03	A	test 1. Brine flow rate.
4	0.240 ± 0.005	0.480 ± 0.005	1.00 ± 0.03	2.00 ± 0.08	0.29 ± 0.08–0.78 ± 0.03	B	test 3. Reactor conf.
5	0.240 ± 0.005	0.480 ± 0.005	1.00 ± 0.03	0.66 ± 0.04	0.24 ± 0.08–0.90 ± 0.03	C	test 1. Reactor conf.
6	0.240 ± 0.005	0.480 ± 0.005	1.00 ± 0.03	2.00 ± 0.08	0.8 ± 0.03–1.20 ± 0.03	C	test 5. Brine flow rate.

a Configuration adopted. Letters A, B, and C refer to Figure 2.

The last column in Table 1 indicates the test case which is considered for comparison purposes and the only parameter being different in the two compared tests. More precisely, as previously mentioned in Section 2.1, one initial objective of the experiments was to assess the influence of the initial Mg^2+^ concentration on the characteristics of the final produced particles. Therefore, as can be observed in Table 1, two different concentrations of Mg^2+^ were taken into consideration in test 1 and test 2 employing the same operating parameters for comparison purposes, for example, a brine flow rate of 0.66 L/min. The two investigated Mg^2+^ concentrations (1 and 0.24 M) are representative of a typical Mg^2+^ concentration of saltwork brines (1 M), which are the objective of the European Project SEArcularMINE,^52^ and a case study of a precise nanofiltration unit (0.24 M), that is, today, part of a novel ZLD treatment chain proposed by the European funded project WATER MINING.^51^ The latter concentration was important to analyze since recent years have seen a greater interest toward nanofiltration as a pre-treatment step for desalination technologies and/or ZLD systems.^53,54^ Flow rate values were chosen in accordance with target prototype flow rates to be adopted in the WATER MINING and SEArcularMINE projects.

The aim of Test 3 was to investigate the influence of brine flow rates, namely, 0.66 L/min (test 1) and 2.00 L/min (test 3), on the obtained Mg(OH)_2_ final product at a fixed Mg^2+^ brine concentration of 0.24 M. Test 4 provided data to be compared to those of test 3 to assess the influence of the A and B reactor configurations (see Figure 2) at a fixed Mg^2+^ concentration of 0.24 M and a brine flow rate of 2.00 L/min. Finally, tests 5 and 6 were carried out by adopting the reactor configuration C. Data of test 5 were compared to those of test 1 to investigate the influence of the recycling strategy on the Mg(OH)_2_ product characteristics by treating a 0.24 M Mg^2+^ solution and adopting a brine flow rate of 0.66 L/min. Furthermore, the comparison between results of tests 5 and 6 was aimed at determining the influence of brine flow rates, namely, 0.66 L/min (test 5) and 2.00 L/min (test 6), when a recycling strategy was adopted by treating 0.24 M Mg^2+^ solutions.

### 2.2 Experimental Procedure

#### 2.2.1 Preparation of Feed and Reactant Solution

The employed brine and alkaline solutions were prepared using deionized water (conductivity below 15 μS/cm). NaOH pellets (technical grade, purity >97%, INOVYN) were used to prepare the alkaline solution. Magnesium chloride hexahydrate (MgCl_2_·6H_2_O) (technical grade, purity >97%, Chem-Lab, Belgium) pellets were employed to prepare the brine solution. Table 1 lists the brine and alkaline solutions prepared for each experimental test. The final compositions of all brine solutions were checked via ion chromatography (Metrohm 882 Compact IC plus). Final compositions of the alkaline solutions were checked via titration.

#### 2.2.2 Sampling Procedure

At the beginning of each experimental run, the reactor was tested in order to analyze its stability. During this initial stage, also known as the “start-up stage”, the flow rates of brine and NaOH solutions were set and monitored by means of the interfacial panel developed in LabVIEW software. Once the values of such parameters were stable (or varied within a very small range, more or less 2%, with respect to the target flow rate value), it was possible to proceed with sampling. As mentioned in Section 2.1, during the experimental test, the flow rate of the alkaline solution was increased in order to reach the desired pH values of 10.1, 10.4, and 12 in the outlet Mg(OH)_2_ slurry. For each value of alkaline solution flow rate, two samples of 1 L of the outlet stream [Mg(OH)_2_ slurry] were taken. One sample was dedicated to sedimentation and filtration analyses (and subsequently to ion chromatography analysis of the filtrate); meanwhile, the second sample was exclusively employed for granulometric analyses. It is worth mentioning that for tests 1 and 2, no sample at pH 10.4 was taken. This was mainly due to difficulties (caused by the combination of low brine flow rates and the adoption of no recycling strategy) encountered within fixing the operating conditions of the reactor.

#### 2.2.3 Analytical Procedure

**Figure 3:**
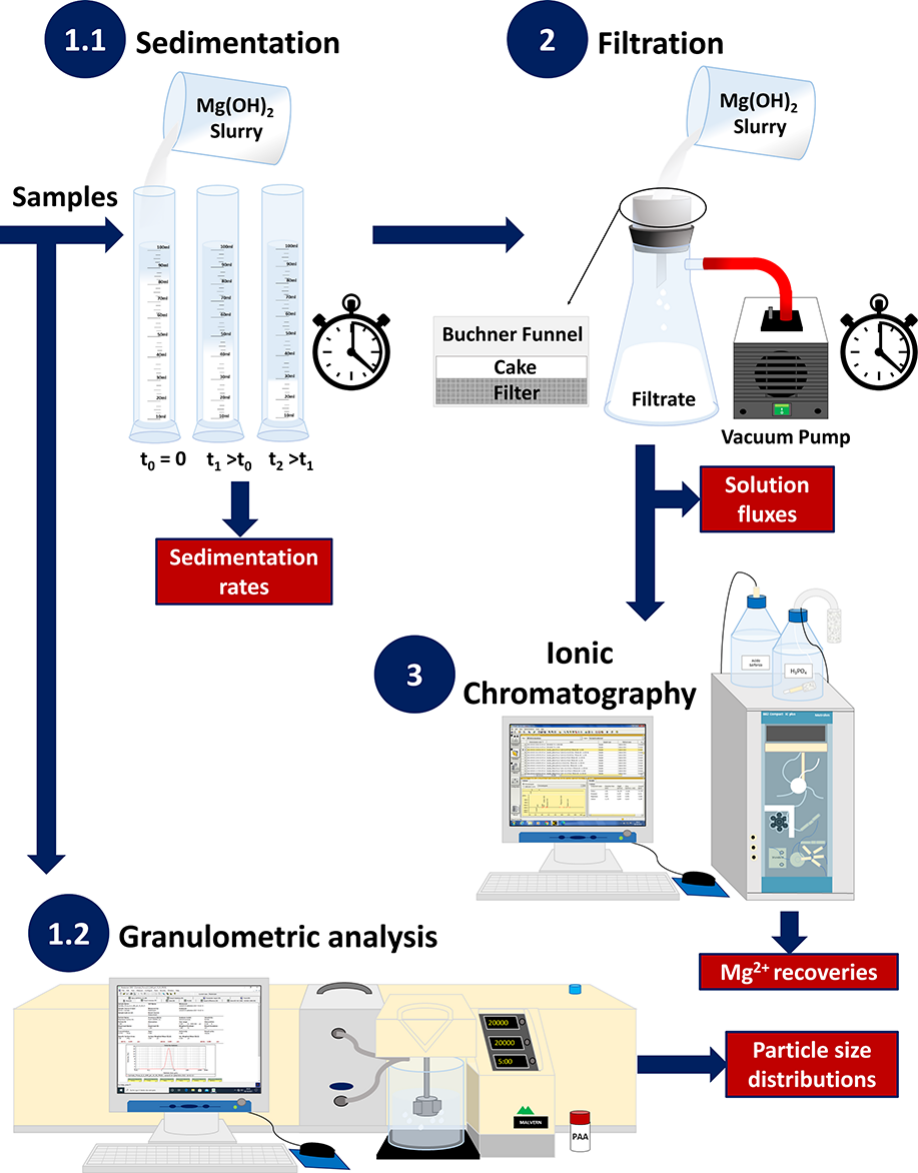
Conceptual scheme of the entire analytical procedure: (1.1) analysis of sedimentation trends in time; (1.2) granulometric analysis via the use of a Malvern Mastersizer 2000; (2) analysis of filtration trends; and (3) analysis of Mg^2+^ recovery via ion chromatography.

Figure 3 illustrates the conceptual scheme of the entire analytical procedure adopted for each produced Mg(OH)_2_ suspension.

Once the experimental run terminated, sedimentation analyses were performed for each Mg(OH)_2_ slurry having different pH values of 10.1, 10.4, and 12 due to the different NaOH flow rates adopted in the reactor. Note that, the final Mg(OH)_2_ slurry pH was not varied after its collection from the reactor outlet. Such analysis was conducted in order to evaluate the sedimentation rate of the Mg(OH)_2_ solid. Each sample was intensely agitated first in order to guarantee that all solids were completely suspended within the sample holder. Second, 100 mL of the sample was poured into a calibrated volumetric glass cylinder, as shown in Figure 3(1.1). At regular time intervals, the volume of the sediment was recorded. Measurements were taken every 30 min at the beginning of the analysis and, toward lower rates of sedimentation, every 1 or 2 h. The duration of each analysis depended on the time required to reach a plateau of the sediment volume measured within time.

Following sedimentation analyses, samples were filtered after re-suspension, as shown in Figure 3(2). To this end, a simple but effective experimental setup was together consisting of (i) a vacuum pump (Buchi V-100), (ii) a 125 mL vacuum flask, (iii) an analog glycerine-filled vacuum pressure gauge to monitor the pressure at which filtration occurs, (iv) a needle valve to adjust the operating pressure of filtration, (v) a Büchner funnel, (vi) glass microfiber filters with a diameter of 70 mm and a pore dimension equal to 1.6 μm (Whatman GF/A grade, GE Healthcare Life Sciences), and (vii) rubber rings to guarantee a mechanical seal between the funnel and the flask. Furthermore, the filtration of 50 mL of sample was performed at a fixed pressure of 0.5 bar by means of the needle valve. Such a pressure was applied for the filtration of the samples of all tests. Once the time of complete filtration was recorded, the solution flux of each sample was calculated (see Section 2.2.4) in order to assess the filterability of the samples.

The filtrate was then analyzed via ion chromatography (882 Compact IC plus, Metrohm) to measure the Mg^2+^ content and assess the Mg^2+^ recovery for each test, as shown in Figure 3(3).

The second remaining sample was destined for granulometric analyses in order to evaluate the produced Mg(OH)_2_ agglomerate/aggregate size distribution, as illustrated in Figure 3(1.2). For such analyses, the static light scattering Malvern Mastersizer 2000 was employed. The granulometer was equipped with a Malvern Hydro 2000 MU that uses a stirrer for the dispersion of the sample into ∼800 mL of deionized water. For each experiment, the stirring velocity was 2000 rpm. Granulometric analyses were carried out as follows: (i) 30 droplets of poly(acrylic acid sodium salt) (PAA, MW: 1200, Sigma-Aldrich, Inc.) were added into the water-filled 800 mL beaker as particles’ dispersant; (ii) 5–40 mL of Mg(OH)_2_ sample, depending on the slurry magma density, was then added in the beaker via Pasteur pipettes until a laser obscuration detected by the apparatus of about 20% was attained; (iii) at least five PSD measurements were performed; afterward, (iv) ultrasound was applied up to a total of 5 min to measure the assemblage state of Mg(OH)_2_ particles and evaluate their fracture strength. Five PSDs were measured after every 5 min of ultrasound treatment. As explained in ref,^55^ the use of a dispersant and sonication are required for the analysis of Mg(OH)_2_ suspensions due to its high flocculation tendency. In fact, only agglomerates made of the actual Mg(OH)_2_ particles would be measured, if they were not broken down.

#### 2.2.4 Definition of Performance Parameters

For the final produced slurry of Mg(OH)_2_ obtained in each experiment, the analysis focused on:

- Sedimentation trend: the profile of the normalized volume fraction of the sedimented Mg(OH)_2_ slurry over time with respect to the total initial volume;
- Cake permeability coefficient: the permeability of the solution across a specific filter cake area normalized with respect to the filter area itself, defined as

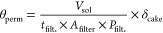
2where θ_perm_ is the permeability coefficient , *V*_sol_ is the volume of solution permeated across the filter cake [L], *t*_filt._ is the time of complete filtration [min], *A*_filter_ is the area of the filter [m^2^], *P*_filt._ is the operating pressure during filtration [bar], and δ_cake_ is the thickness of cake formed during filtration [m] calculated as
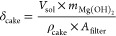
3where  is the magma density of Mg(OH)_2_ slurry [g/L] and ρ_cake_ (assumed to be equal to the water density) is the density of the cake formed during filtration [g/m^3^];

- Mg^2+^ recovery (Y) that accounts for the amount of Mg^2+^ ions recovered from the brine due to the precipitation. It is computed as the ratio of the difference in Mg^2+^ moles in the feed and those in the filtrate with respect to the Mg^2+^ moles in the feed [%];
- magma density of Mg(OH)_2_ slurry calculated as the ratio of the mass of Mg(OH)_2_ solid present in the slurry over the volume of produced slurry [g/L];
- PSDs without and with treatment of the samples by sonication.

In order to understand the impact of the fluid dynamics of the brine and NaOH solutions on Mg(OH)_2_ particles, the Reynolds number within (i) the nozzle and (ii) a section of the bulk mixing zone immediately after the nozzle (where the chemical reaction takes place) was calculated for all the investigated cases as
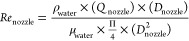
4
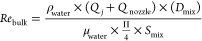
5where *Re*_nozzle_ and *Re*_bulk_ are the Reynolds number within the nozzle and the section of the bulk mixing zone, respectively, *Q*_*j*_ is the flow rate of the host solution receiving the injected one [m^3^/s], *Q*_nozzle_ is the flow rate within the nozzle [m^3^/s], *D*_mix_ is the characteristic diameter of the mixing zone [m], *S*_mix_ is the cross section of the mixing zone [m^2^], and *D*_nozzle_ is the diameter of the nozzle [m]. For the sake of simplicity, the density ρ_water_ [kg/m^3^] and dynamic viscosity μ_water_ [Pa s] of water (0.0082 Pa s and 997 kg/m^3^, respectively, at 20 °C) were considered rather than those of the suspensions. Reynolds number within the nozzle and within the bulk mixing zone of the MF-PFR for all experimental tests are listed in Table 2.

**Table 2:** Nominal Flow Rates and Values of Reynolds Number in the Nozzle and in the Bulk for Experimental Tests

test	slurry pH	*Q*_brine_[L/min]	*Q*_NaOH_[L/min]	*Re*_nozzle_	*Re*_bulk_
1	10.1	0.66	0.12	880	210
	12		0.38		330
2	10.1	0.66	0.59	880	420
	12		1.10		660
3	10.1	2.00	0.38	2650	640
	10.4		0.60		740
	12		0.80		830
4	10.1	2.00	0.29	390	990
	10.4		0.50	660	1040
	12		0.78	1040	1100
5	10.1	0.66	0.24	880	18610
	10.4		0.44		
	12		0.90		
6	10.1	2.00	0.80	2650	18920
	10.4		0.90		
	12		1.20		

## 3 Results and Discussion

### 3.1 Effect of Mg^2+^ Brine Concentration (Configuration A)

Initially, the Mg(OH)_2_ suspensions produced in the operating configuration A (see Figure 2) at different Mg^2+^ initial concentrations were investigated by comparing results of test 1 and test 2 (see Table 1). In configuration A, the brine was fed through distributed nozzles toward the NaOH solutions flowing in the adjacent compartment. Solutions mixed as brine were injected into the NaOH solution. As reported in Section 2.2.2, at pH 10.4, no sample was taken during test 1 and test 2 due to difficulties encountered within fixing the operating conditions of the reactor.

Reynolds number was calculated for both tests within the bulk of the mixing zone *Re*_bulk_ and inside the nozzle *Re*_nozzle_ (see Table 2). Tests are characterized by the same brine flow rate and therefore the same *Re*_nozzle_, while *Re*_bulk_ varied due to the higher NaOH flow rate (see Table 3) employed in the case of 1 M concentration (to ensure a stoichiometric mole flow of Mg^2+^ and OH^–^ ions).

**Table 3:** *Re*_nozzle_, Magma Density, and Mg^2+^ Recovery for Tests 1 and 2

	slurry pH	*Q*_brine_[L/min]	*Q*_NaOH_[L/min]	*Re*_nozzle_	magma density [g/L]	Mg^2+^ recovery [%]
1	10.1	0.66	0.12	880	5.56	46.9
	12		0.38		9.65	93.3
2	10.1	0.66	0.59	880	27.1	88.1
	12		1.10		21.6	98.7

**Figure 4:**
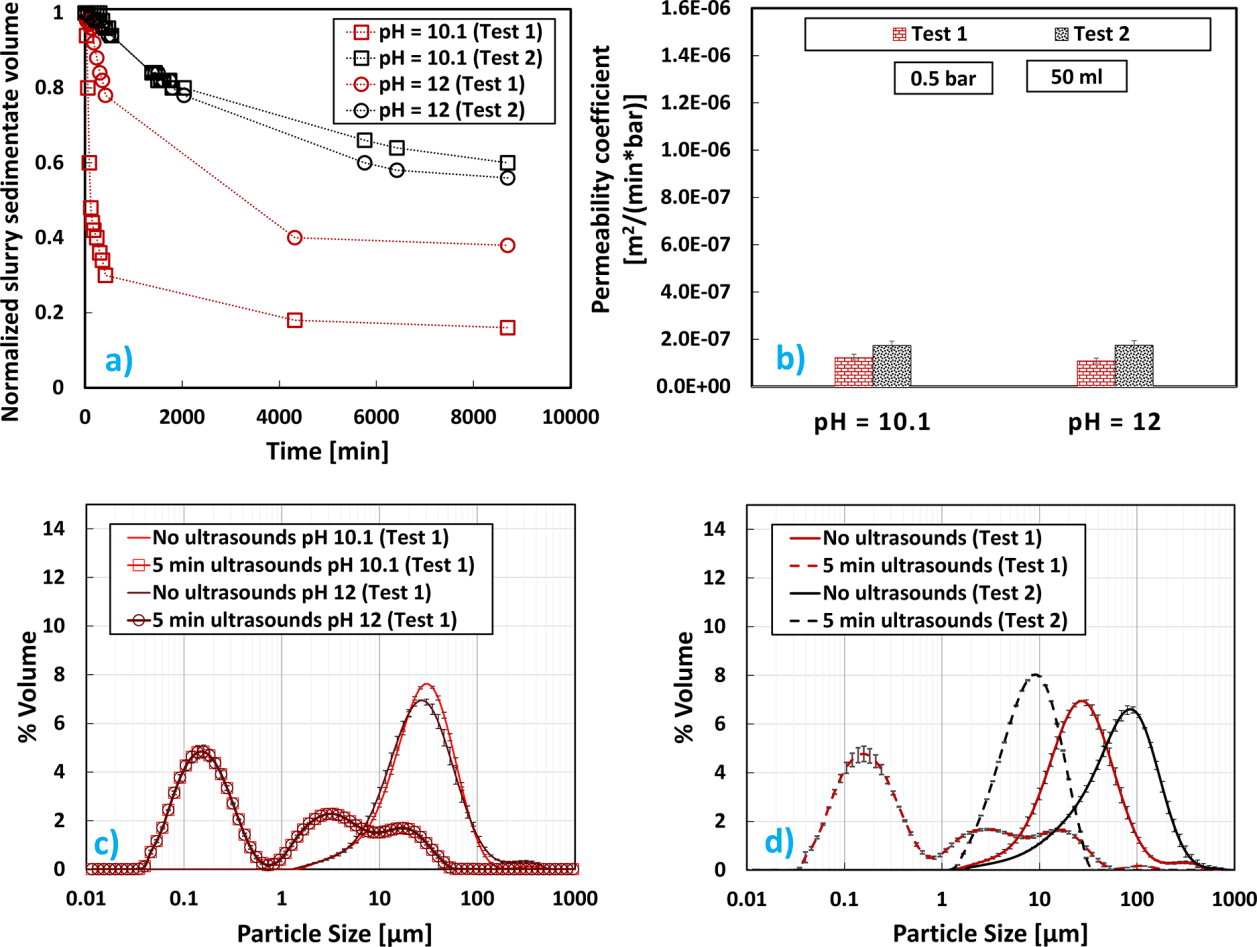
Mg(OH)_2_ results at pH values 10.1 and 12 for initial Mg^2+^ concentrations of 0.24 M (test 1) and 1 M (test 2): (a) sedimentation trend over time; (b) cake permeability coefficients; (c) V-PSDs (effect of pH); (d) V-PSDs (comparison between tests 1 and 2 at pH = 12).

Figure 4a reports the sedimentation trend of the normalized volume of Mg(OH)_2_ slurry over time [*V*(t)/*V*_initial_] for test 1 and test 2 at two different pH values: 10.1 and 12.

As can be observed, Mg(OH)_2_ suspensions produced from a lower Mg^2+^ feed concentration (test 1, red lines and symbols in Figure 4a) sedimented more quickly than those at a higher concentration (test 2, black lines and symbols in Figure 4a). This was expected as the slurry magma density was lower for test 1 than test 2 (see Table 3). Furthermore, another important reason for this behavior lies within the higher supersaturation condition reached with a higher Mg^2+^ concentration. More precisely, higher supersaturation leads to the formation of smaller particles that form agglomerates that entrap a higher mother liquor amount producing more isodense particles, thus causing slower sedimentation rates at 1 M.^42^ In order to assess the filterability of the product, the cake permeability coefficient (eq 2) was calculated and compared for test 1 and test 2, Figure 4b. As can be observed, the cake permeability of the product was not greatly influenced by the initial different Mg^2+^ concentration of the feed solution. Lower Mg^2+^ concentrations were characterized by slightly lower permeabilities due to the lower magma density of the filtered suspension. Furthermore, a slight decrease of permeability was noted when increasing the slurry pH from 10.1 to 12. As expected, at pH 10.1, Mg^2+^ ions were only partially converted in Mg(OH)_2_. Conversely, the extent of the conversion was found to be dependent on the initial Mg^2+^ concentration: Mg^2+^ recovery rates in test 1 were equal to 46.9 and 93.3%, while in test 2, the rates were 88.1 and 98.7% (respectively, at pH 10.1 and 12), as reported in Table 3. Figure 4c illustrates the volume PSDs (V-PSDs) of the Mg(OH)_2_ particles obtained before and after sonication for test 1 at different pH values (pH 10.1 and 12). It was observed that the slurry pH had no influence whatsoever on the V-PSDs. For such reason, the comparison of V-PSDs of different tests will be shown at the same slurry pH (pH 12) throughout the paper. Figure 4d illustrates the V-PSDs of the Mg(OH)_2_ particles obtained for test 1 and test 2 only for the pH value of 12 before and after sonication. Similar V-PSDs were obtained when no sonication was applied. In particular, particle sizes ranged between 1–100 μm and 1–500 μm for test 1 and 2, respectively. V-PSDs considerably differed after sonication. As a matter of fact, larger agglomerates (1–20 μm size) were obtained at a high concentration (test 2), with respect to the smaller aggregates/agglomerates detected for test 1, most of the particles being in the sizes range between 0.08 and 1 μm. This was because the lower concentration of the feed brine in test 1, despite the higher Reynolds regime in test 2 with consequently higher mixing intensity, resulted in a more uniform distribution of the local supersaturation. As a matter of fact, the Mg(OH)_2_ precipitation process rate was slower in test 1 due to the lower Mg^2+^ concentration that was almost four times lower than that in test 2. In such conditions, better mixing of the reactants led to a more homogeneous supersaturation level for the Mg(OH)_2_ reactive crystallization that produced weak agglomerates of nanosized aggregates that can be easily broken down. On the other hand, stronger agglomerates of nanosized aggregates were formed in test 1 that require high energy to be broken, as discussed by Battaglia et al.^55^

### 3.2 Effect of Brine Flow Rate (Configuration A)

**Figure 5:**
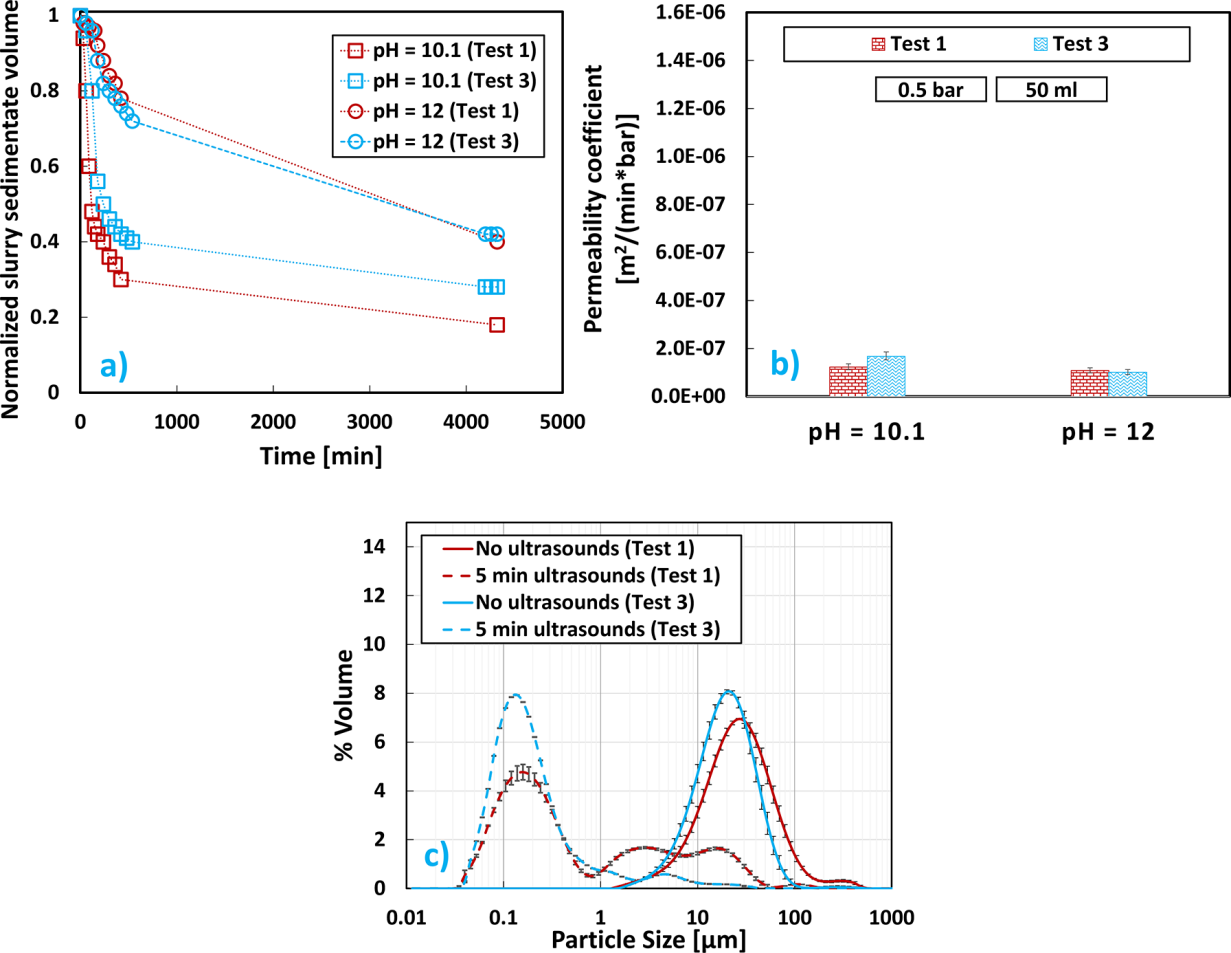
Mg(OH)_2_ results at pH values of 10.1 and 12 for brine flow rates equal to 0.66 L/min (test 1) and 2 L/min (test 3): (a) sedimentation trend over time; (b) cake permeability coefficients; (c) V-PSDs.

Adopting the same operating configuration A as that in Section 3.1 (Figure 2a), it was investigated how the brine flow rate in the distribution section could influence the final product. At a fixed Mg^2+^ concentration equal to 0.24 M, two different brine flow rates were then studied: 0.66 and 2 L/min (test 1 and test 3, see Table 1). Such values led, in this case, to different *Re*_nozzle_ values within the nozzle, as reported in Table 4. As described in Section 2.2.2, at pH 10.4, no sample was taken during test 1 due to difficulties encountered when fixing the operating conditions of the reactor. Figure 5a reports the sedimentation trend for test 1 and test 3 at two different pH values: 10.1 and 12.

**Table 4:** *Re*_nozzle_, Magma Density, and Mg^2+^ Recovery for Tests 1 and 3

	slurry pH	*Q*_brine_[L/min]	*Q*_NaOH_[L/min]	*Re*_nozzle_	magma density [g/L]	Mg^2+^ recovery [%]
1	10.1	0.66	0.12	880	5.56	46.9
	12		0.38		9.79	93.3
3	10.1	2.00	0.38	2650	6.89	58.6
	12		0.80		9.55	95.5

As can be observed, the brine flow rate did not have much influence whatsoever on the suspension sedimentation as very similar trends were observed. This could be attributed to the fact that the obtained Mg(OH)_2_ suspensions were very similar to each other being characterized by close magma densities and almost equal PSDs. Once again suspensions at higher pH values sedimented slower than those at low pH. As for the filterability of the final product, very similar cake permeability coefficients were obtained at different operating brine flow rates. As seen in Section 3.1, also in this case, a decrease of the permeability coefficient was observed when increasing the slurry pH value. Interestingly, when comparing test 1 with test 3, the increased mixing of test 3 had a noticeable effect only at the lower pH of 10.1, where a slower settling but a higher permeability was obtained (underlining the opposite trend of these two parameters), along with a higher Mg^2+^ recovery (58.6% against 46.9%, respectively, for test 1 and test 3), whereas negligible differences in every parameter analyzed during test 1 and test 3 were achieved at pH = 12 (see Table 4). Figure 5c reports the V-PSD, before and after sonication, of the Mg(OH)_2_ particles obtained for tests 2 and 3 at slurry pH of 12, since no difference in the PSDs was observed at different pH values. As can be observed, before sonication, different brine flow rates did not lead to different initial V-PSDs, since they referred to Mg(OH)_2_ agglomerates. After sonication, V-PSDs were centered around the order of magnitude of nanometers. However, it could be noted how lower brine flow rates in test 1 and therefore lower Reynolds values and mixing degree in the reactor led to a mixture of aggregates and agglomerates of particles characterized by diameters in the range of nanometers and micrometers. Conversely, at the higher mixing condition of test 3, almost no micrometer-sized agglomerates could be observed. It is therefore possible to conclude that operating at higher flow rates allows achieving a tighter unimodal final V-PSD of the final product, centered always more toward the order of magnitude of nanometers.

### 3.3 Effect of the Hydrodynamic Asset (Configurations A vs B)

**Figure 6:**
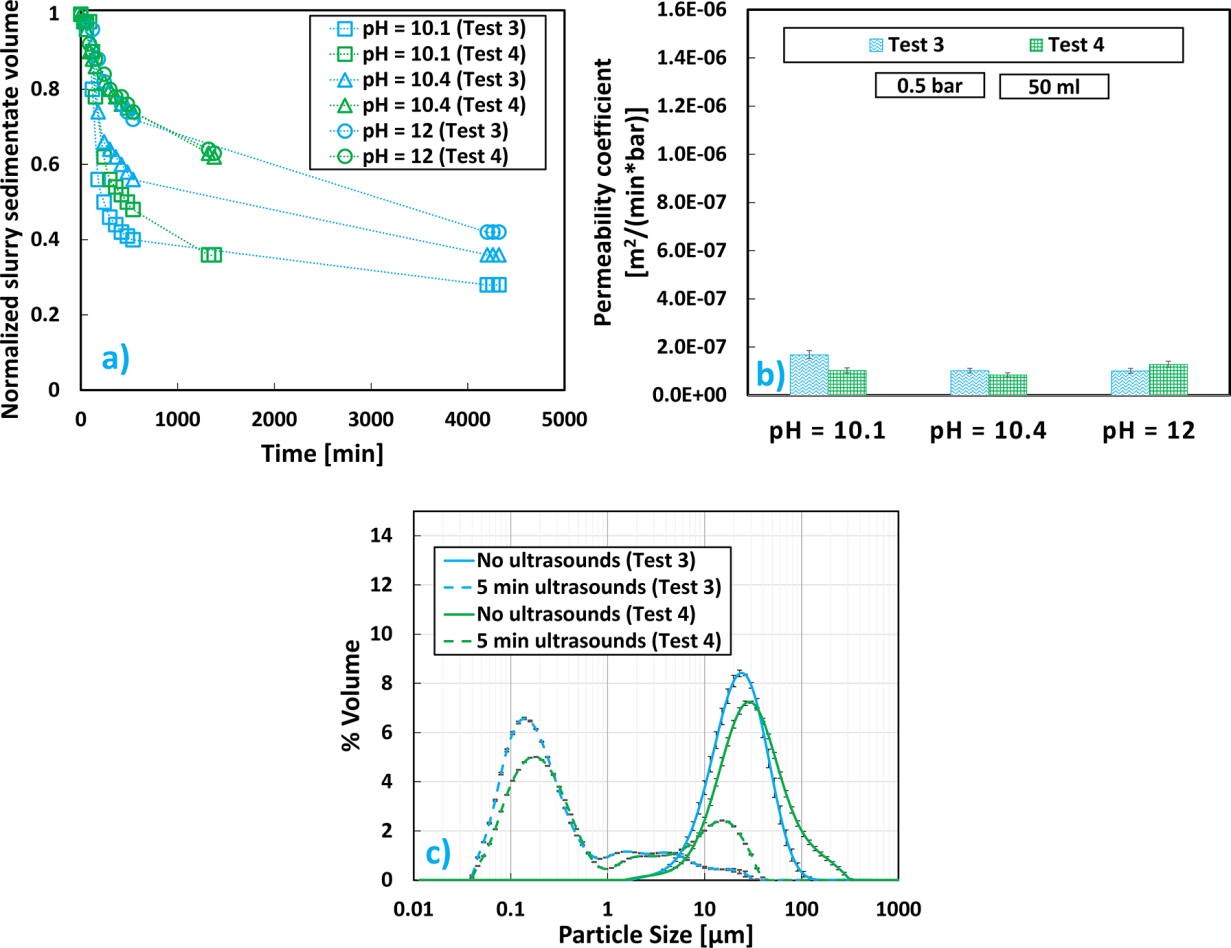
Mg(OH)_2_ results at pH values 10.1, 10.4, and 12, brine flow rate equal to 2 L/min, and initial Mg^2+^ concentration of 0.24 M for configuration A (test 3) and configuration B (test 4): (a) sedimentation trend over time; (b) cake permeability coefficients; (c) V-PSDs.

The MF-PFR was initially designed in such a way that the brine would enter a distribution section and, by means of nozzles, be injected into the alkaline solution in the adjacent section (configuration A of Figure 2). To fully investigate the MF-PFR capabilities, it was tested what could occur and the effects on the final products when switching the brine and NaOH feed position, respectively (configuration B, see Figure 2b). In this way, the alkaline reactant was injected into the brine solution. A comparison of produced Mg(OH)_2_ suspensions was then carried out, comparing test 3 and test 4 (see Table 1). As can be seen in Figure 6a,b, when the feed streams were switched, a switch in sedimentation trends and filtration trends was also achieved.

It is clear for the lowest pH value of 10.1 where a slower settling of test 4 also corresponded to a smaller cake permeability when compared to test 3. While, at pH 10.4, the behavior was more or less similar to the one at pH 10.1; at pH 12, test 4 outperformed the performance of test 3 in both the settling rate and permeability of the cake. This could be interpreted by observing the behavior of the Reynolds numbers both for the bulk (Table 2) and the nozzle (Table 5). *Re*_bulk_ of test 4 was always greater than *Re*_bulk_ of test 3, but it was only at pH 12 that *Re*_nozzle_of test 4 became of the same order of magnitude of test 3. The lower *Re*_nozzle_for test 4 than test 3, due to less powerful jets shot into the host solution via the nozzles, led to poorer control of the final V-PSD, as can be observed in Figure 6c. This result could be attributed to an inhomogeneous supersaturation of the bulk that caused the production of a greater mixture of different particle sizes. As a matter of fact, a narrower peak was achieved when applying ultrasound to the sample produced for test 3.

**Table 5:** *Re*_nozzle_, Magma Density, and Mg^2+^ Recovery for Tests 3 and 4

	slurry pH	*Q*_brine_[L/min]	*Q*_NaOH_[L/min]	*Re*_nozzle_	magma density [g/L]	Mg^2+^ recovery [%]
3	10.1	2.00	0.38	2650	6.89	58.6
	10.4		0.60		8.31	77.2
	12		0.80		9.55	95.5
4	10.1	2.00	0.29	390	6.30	51.6
	10.4		0.50	660	7.91	70.7
	12		0.78	1040	9.42	93.6

### 3.4 Effect of Mg(OH)_2_ Suspension Recirculation (Configuration C)

**Figure 7:**
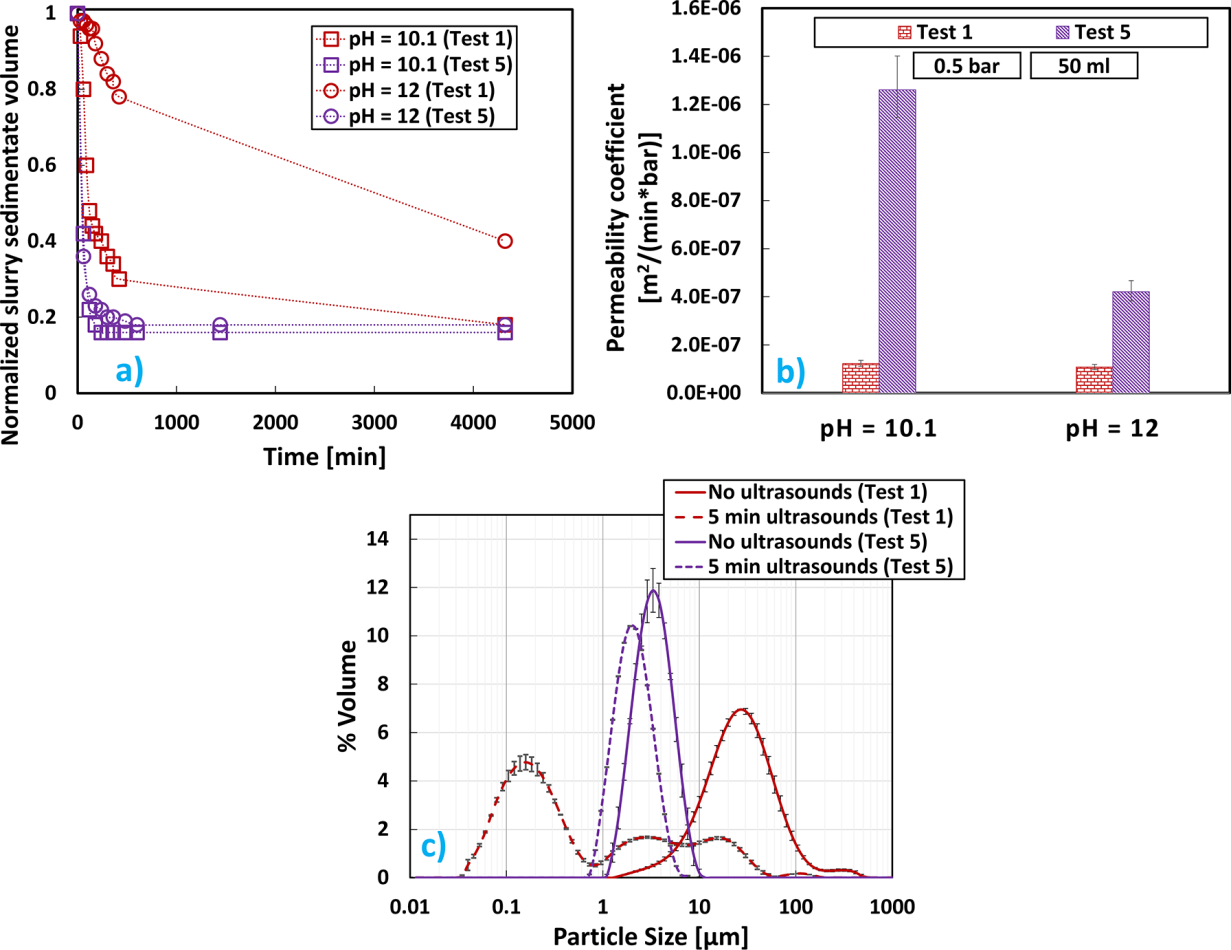
Mg(OH)_2_ results at pH values 10.1 and 12, brine flow rate equal to 0.66 L/min, and initial Mg^2+^ concentration of 0.24 M for configuration A (test 1) and configuration C (test 5): (a) sedimentation trend over time; (b) cake permeability coefficients; (c) V-PSDs.

The configuration C (Figure 2c) of the MF-PFR was also tested. Such configuration consisted in partially mixing the Mg(OH)_2_ slurry that exits the reactor with fresh alkaline solution and resending it back to the inlet of the MF-PFR. As described in Section 2.2.2, at pH 10.4, no sample was taken during test 1 due to difficulties encountered within fixing the operating conditions of the reactor. In terms of bulk Reynolds number, the recycling strategy significantly increased such value (see Table 2). Considerable differences were noticed with this new configuration, as can be seen in Figure 7.

Mg(OH)_2_ suspensions that proceeded using the recycling strategy (test 5) sedimented much faster than those when no recycling was adopted (test 1). It is interesting to note that, for test 5, there was no influence of pH on the sedimentation process of Mg(OH)_2_ suspensions, in contrast with all the results reported in Sections 3.1, 3.2, and 3.3. However, the influence of pH on cake permeability was apparent, where at pH 12 it was less than a half of that at pH 10.1 in test 5, while this difference was barely appreciable for test 1. When test 5 permeability was compared with that of test 1, it could be seen how adopting the recirculation strategy resulted in an increase of almost one order of magnitude with respect to suspensions produced without recycling. Both behaviors (faster sedimentation and filtration) are for sure of great interest for industrial applications. Furthermore, as can be observed in Figure 7c, V-PSDs of test 5 slightly changed after ultrasound treatment, always showing a peak between 1 and 10 μm that could be correlated to stronger agglomerates induced by the recycling strategy. This was confirmed by the faster sedimentation and filtration shown in Figure 7a,b. It was also interesting to observe how the recycling strategy was able to offer another advantage when compared to configuration A. Such advantage consisted in the possibility of achieving 100% recovery at pH 12 (test 5) unlike the 93.3% recovery obtained with test 1 (see Table 6).

**Table 6:** *Re*_nozzle_, Magma Density, and Mg^2+^ Recovery for Tests 1 and 5

	slurry pH	*Q*_brine_[L/min]	*Q*_NaOH_[L/min]	*Re*_nozzle_	magma density [g/L]	Mg^2+^ recovery [%]
1	10.1	0.66	0.12	880	5.56	46.9
	12		0.38		9.79	93.3
5	10.1	0.66	0.24	880	6.75	65.8
	12		0.90		5.54	100

### 3.5 Effect of Brine Flow Rate (Configuration C)

**Figure 8:**
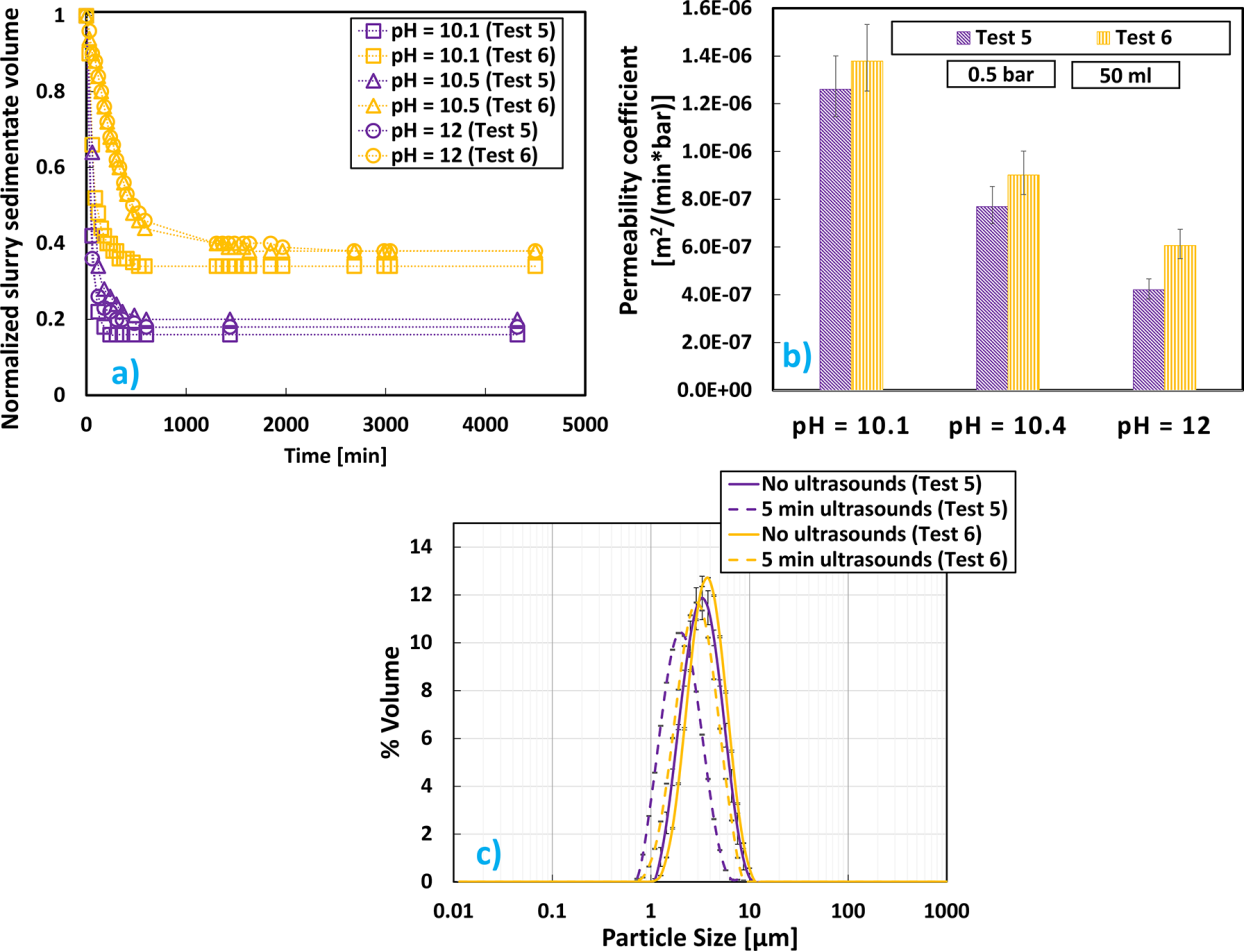
Mg(OH)_2_ results at pH values 10.1, 10.4, and 12 for brine flow rates equal to 0.66 L/min (test 5) and 2 L/min (test 6): (a) sedimentation trend over time; (b) cake filtration permeability coefficients; (c) V-PSDs.

Adopting the same recycling strategy of Section 3.4, in which the Mg(OH)_2_ slurry produced is partially mixed with fresh alkaline solution and sent back to the reactor, the brine flow rate on the final product effect was analyzed. Increasing the brine flow rate confirmed the previous finding where the slower settling recorded in test 6 corresponded to a larger permeability (see Figure 8). Again, the higher the pH, the lower the permeability, for both test 5 and test 6. As mentioned previously, the recycling strategy allowed the control of the size distribution of the final product, obtaining a V-PSD centered at 4–5 μm. Furthermore, as reported in Table 7, the adoption of the recycle strategy had a considerable influence on the Mg^2+^ recovery at a pH value of 10.4. As a matter of fact, a 100% recovery was achieved with respect to the highest value of 77% achieved in test 3 at the same pH value. This was due to the use of the recycle of the outlet stream and its mixing with fresh NaOH solution in the recycle stream, as described in Section 2.

**Table 7:** *Re*_nozzle_, Magma Density, and Mg^2+^ Recovery for Tests 5 and 6

	slurry pH	*Q*_brine_[L/min]	*Q*_NaOH_[L/min]	*Re*_nozzle_	magma density [g/L]	Mg^2+^ recovery [%]
5	10.1	0.66	0.24	880	6.75	65.7
	10.4		0.44		7.60	100
	12		0.90		5.54	100
6	10.1	2.00	0.80	2650	8.52	85.2
	10.4		0.90		9.65	100
	12		1.20		8.75	100

## 4 Conclusions

The Mg(OH)_2_ precipitation process from synthetic solutions was studied by adopting a novel MF-PFR crystallizer, purposely designed for the production of Mg(OH)_2_ at a pilot scale. Different reactor assets were investigated addressing the influence of (i) initial Mg^2+^ concentrations, mimicking the ones of waste Mg-rich solutions of saltwork bitterns (Mg^2+^ 1.0 M) and wastewater treatment plants (Mg^2+^ 0.24 M); (ii) different reactant flow rates; and (iii) adopting a product recycling strategy (seeded crystallization).

(i) A higher initial Mg^2+^ concentration (1.0 M) of the feed brine led to the production of larger and stronger agglomerates of Mg(OH)_2_ particles than those produced by a lower initial concentration (0.24 M). In particular, after the application of ultrasound and adoption of a dispersant agent, microsized Mg(OH)_2_ agglomerates/aggregates were measured in the case of 1.0 M Mg^2+^ solutions, while nanosized and microsized particles were detected for the 0.24 M case.
(ii) No significant influences were observed on the sedimentation trends, filtration times, and granulometry of the final product when different reactant flow rates were employed regardless of the reactor configurations. On the other hand, it was found that, in most of the cases, Mg(OH)_2_ suspensions produced using over-stoichiometric NaOH amounts, final suspension pH 12, were characterized by lower sedimentation rates and cake permeability coefficient values.
(iii) A key aspect was the adoption of a product recycling strategy that favored a seeded crystallization process. Specifically, (a) Mg(OH)_2_ suspensions sedimented up to 4 times faster than those produced without product recycling and (b) the cake permeability coefficient increased, reaching values of up to 1 order of magnitude higher than those of suspensions produced without product recycling.

Overall, the recycling strategy (iii) represents a crucial parameter that can be of considerable importance in order to overcome filterability and sedimentation issues in the large-scale production of Mg(OH)_2_ suspensions, especially for those precipitated using NaOH solutions.

## Nomenclature and acronyms

*A*: area [m^2^]
Ca(OH)_2_: calcium hydroxide
CRM: critical raw material
δ: width [m]
*D*: diameter [m]
HDPE: high-density polyethylene
MF-PFR: multiple feed-plug flow reactor
Mg^2+^: magnesium ion
MgCl_2_: magnesium chloride
Mg(OH)_2_: magnesium hydroxide
*m*: magma density [g/L]
NaOH: sodium hydroxide
NH_4_OH: ammonium hydroxide
θ: permeability coefficient
*P*: operating pressure [bar]
PVC: polyvinyl chloride
*Q*: flow rate [m^3^/s]
*Re*: Reynolds number [—]
ρ: density [g/m^3^]
*S*: cross section [m^2^]
*t*: time [min]
μ: dynamic viscosity [Pa s]
V-PSD: volume particle size distribution
*V*: volume [L]
Y: magnesium recovery [%]
ZLD: zero liquid discharge

## Suffixes

aq: liquid state of aggregation
bulk: bulk mixing zone
filt: filtration
*i*: solution within the nozzle
*j*: host solution receiving the injected one
Mg(OH)_2_: magnesium hydroxide
mix: mixing zone
nozzle: nozzle zone
perm: permeability
*s*: solid state of aggregation
sol: solution permeated across the filter cake
